# Neuroacanthocytosis Syndromes

**DOI:** 10.1186/1750-1172-6-68

**Published:** 2011-10-25

**Authors:** Hans H Jung, Adrian Danek, Ruth H Walker

**Affiliations:** 1 Department of Neurology, University Hospital Zürich, Zürich, Switzerland; 2 Department of Neurology, Ludwig-Maximilians-Universität, München, Germany; 3 Department of Neurology, Veterans Affairs Medical Center, Bronx, NY, USA

## Abstract

Neuroacanthocytosis (NA) syndromes are a group of genetically defined diseases characterized by the association of red blood cell acanthocytosis and progressive degeneration of the basal ganglia. NA syndromes are exceptionally rare with an estimated prevalence of less than 1 to 5 per 1'000'000 inhabitants for each disorder. The core NA syndromes include autosomal recessive chorea-acanthocytosis and X-linked McLeod syndrome which have a Huntington´s disease-like phenotype consisting of a choreatic movement disorder, psychiatric manifestations and cognitive decline, and additional multi-system features including myopathy and axonal neuropathy. In addition, cardiomyopathy may occur in McLeod syndrome. Acanthocytes are also found in a proportion of patients with autosomal dominant Huntington's disease-like 2, autosomal recessive pantothenate kinase-associated neurodegeneration and several inherited disorders of lipoprotein metabolism, namely abetalipoproteinemia (Bassen-Kornzweig syndrome) and hypobetalipoproteinemia leading to vitamin E malabsorption. The latter disorders are characterized by a peripheral neuropathy and sensory ataxia due to dorsal column degeneration, but movement disorders and cognitive impairment are not present. NA syndromes are caused by disease-specific genetic mutations. The mechanism by which these mutations cause neurodegeneration is not known. The association of the acanthocytic membrane abnormality with selective degeneration of the basal ganglia, however, suggests a common pathogenetic pathway. Laboratory tests include blood smears to detect acanthocytosis and determination of serum creatine kinase. Cerebral magnetic resonance imaging may demonstrate striatal atrophy. Kell and Kx blood group antigens are reduced or absent in McLeod syndrome. Western blot for chorein demonstrates absence of this protein in red blood cells of chorea-acanthocytosis patients. Specific genetic testing is possible in all NA syndromes. Differential diagnoses include Huntington disease and other causes of progressive hyperkinetic movement disorders. There are no curative therapies for NA syndromes. Regular cardiologic studies and avoidance of transfusion complications are mandatory in McLeod syndrome. The hyperkinetic movement disorder may be treated as in Huntington disease. Other symptoms including psychiatric manifestations should be managed in a symptom-oriented manner. NA syndromes have a relentlessly progressive course usually over two to three decades.

## Definition

**Figure 1:**
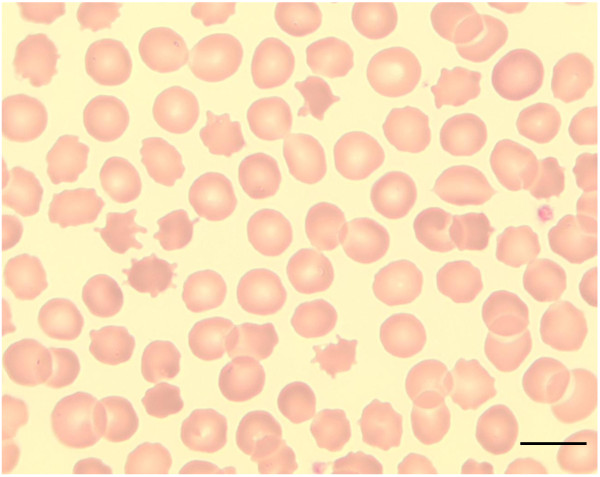
**Acanthocytes**. Peripheral blood smear showing acanthocytosis in a patient with McLeod syndrome (May Gruenwald-Giemsa; x100; scale bar = 10 μm).

Neuroacanthocytosis (NA) refers to a heterogeneous group of syndromes in which nervous system abnormalities coincide with red blood cell acanthocytosis, i.e. deformed erythrocytes with spike-like protrusions (Figure 1) [1]. However, acanthocytosis can be variable, and the diagnosis of these syndromes does not require their demonstration on peripheral blood smear. There are two broad groups of NA disorders (Table 1). First, the so-called "core" NA syndromes characterized by degeneration of the basal ganglia, movement disorders, cognitive impairment and psychiatric features, and second, conditions with alteration of lipoprotein metabolism, namely abetalipoproteinemia (Bassen-Kornzweig syndrome) and hypobetalipoproteinemia leading to vitamin E malabsorption, with the clinical hallmarks of peripheral neuropathy and sensory ataxia due to dorsal column degeneration, but without movement disorders. In addition, there are several sporadic conditions associated with acanthocytosis (Table 1). This review will refer only to the first group of NA syndromes.

**Table 1:** Neuroacanthocytosis syndromes

Core neuroacanthocytosis syndromes	Neuroacanthocytosis with lipoprotein disorders	Acanthocytosis in systemic diseases where neurological findings may also be present
Chorea-acanthocytosis (ChAc)	Abetalipoproteinemia (Bassen-Kornzweig syndrome)	Severe malnutrition (e.g. anorexia nervosa)
McLeod syndrome (MLS)	Familial hypobetalipoproteinemia	Cancers, sarcoma
Huntington's disease-like 2 (HDL2)	Anderson disease	Thyroid disorders, myxoedema
Pantothenate kinase associated neurodegeneration (PKAN)	Atypical Wolman disease	Splenectomy
		Liver cirrhosis, hepatic encephalopathy
		MELAS
		Psoriasis
		Eales' disease (angiopathia retinae juvenilis)

MELAS, mitochondrial encephalopathy with lactic acidosis and stroke-like episodes.

NA syndromes were known initially under the eponym "Levine-Critchley syndrome" [2,3]. The clinical description of the subjects reported by Critchley is compatible with autosomal recessive chorea-acanthocytosis (ChAc; ORPHA2388) and preliminary genetic data from the index family support this diagnosis. However, the inheritance pattern and clinical features of the family described by Levine is not fully compatible with either ChAc or with the X-linked recessive McLeod syndrome (MLS; ORPHA59306). A retrospective genetic analysis so far has not been possible, as this family unfortunately appears lost to follow-up, and thus the original eponym appears obsolete.

MLS was named after a Harvard dental student, Hugh McLeod, in whom an abnormal erythrocyte antigen pattern (absent or weak expression of Kell antigens) was first described [4]. Initially, the McLeod blood group phenotype was thought to be of no clinical significance, apart from the requirement for matched blood transfusions. Later it was found that the McLeod blood group phenotype was also observed in boys with X-linked chronic granulomatous disease (CGD; ORPHA379) [5] and that asymptomatic adult male carriers of the McLeod blood group phenotype have elevated serum levels of CK reflecting muscle cell pathology [6]. Subsequently it was recognized that McLeod carriers had a "neurological disorder characterized by involuntary dystonic or choreiform movements, areflexia, wasting of limb muscles, elevated CK, and congestive cardiomyopathy", thus defining MLS as a multi-system disorder with hematological, neuromuscular, and central nervous system (CNS) involvement [7].

In 1991, Hardie and colleagues described a series of 19 NA patients, which for years was the seminal work on NA [8]. However, with recognition of the molecular basis of the different NA syndromes, this case series has turned out to be heterogeneous, including patients with ChAc, MLS and pantothenate kinase-associated neurodegeneration (PKAN; ORPHA157850).

The "core" NA syndromes are now defined as autosomal recessive ChAc caused by mutations of the *VPS13A *gene [9,10], and X-linked MLS, caused by mutations of the *XK *gene [11]. Additionally there are several genetically defined disorders in which acanthocytosis is occasionally seen, such as PKAN [12] and Huntington disease-like 2 (HDL2; ORPHA98934) [13]. Occasional rare cases or families are reported where acanthocytes are present in concert with other extrapyramidal features, such as paroxysmal dyskinesias [14] or mitochondrial disease [15].

## Epidemiology

NA disorders are all exceedingly rare, but also very likely to be underdiagnosed. Estimates suggest that there are probably around one thousand ChAc cases and a few hundred cases of MLS worldwide. ChAc appears to be more prevalent in Japan, possibly due to a genetic founder effect [10], and clusters have been found elsewhere in geographically isolated communities, e.g. in the French-Canadian population [16]. MLS has been described in Europe, North and South America, and Japan without obvious clustering [17]. PKAN is somewhat more common with an estimated prevalence of 1 to 3/1'000'000. HDL2 is very rare, with less than 50 families identified worldwide. The vast majority of families are of African ancestry [18], including two Brazilian families in whom the African ethnic background was not apparent on initial examination [19]. One HDL2 case has been reported of "middle Eastern" origin [20], but further details are not available.

## Clinical Characteristics

The core NA syndromes ChAc and MLS have a Huntington disease-like phenotype with an involuntary hyperkinetic movement disorder, psychiatric manifestations and cognitive alterations, thus representing phenocopies of HD. Both disorders have an adult onset and a slow progression. However, there are several phenotypic peculiarities, in particular the neuromuscular involvement reflected in signs of myopathy and absent tendon reflexes that allow a clinical suspicion of these two disorders. In addition, hepatosplenomegaly can be seen in both syndromes due to increased hemolysis. HDL2 and PKAN, by contrast, have a childhood or juvenile onset, and HDL2 is usually found in patients with African ancestry.

### Chorea-Acanthocytosis

ChAc is a progressive autosomal recessive neurodegenerative disorder with onset of neurological symptoms usually in the twenties, thus representing a late onset for an autosomal recessive disorder (Table 2) [1]. Often the initial presentation may be subtle cognitive or psychiatric symptoms, and in retrospect patients may have developed related psychiatric complaints several years before the neurological manifestations. Administration of neuroleptics for psychiatric disease may confound the recognition of the movement disorder as due to a neurodegenerative process. In some cases, seizures may precede the appearance of movement disorders by as much as a decade [21]. During the disease course, most patients develop a characteristic phenotype including chorea, a very peculiar "feeding dystonia" with tongue protrusion [22], orofacial dyskinesias, involuntary vocalizations, dysarthria and involuntary tongue- and lip-biting. The gait of ChAc patients may have a "rubber man" appearance with truncal instability and sudden, violent trunk spasms [23]. Most ChAc patients develop generalized chorea and a minority of ChAc patients develops Parkinsonism. In addition to orofaciolingual dystonia, limb dystonia is common. In at least one third of patients, seizures, typically generalized, are the first manifestation of disease. Impairment of memory and executive functions is frequent, although not invariable. Psychiatric manifestations are common and may present as schizophrenia-like psychosis or obsessive-compulsive disorder. Most ChAc patients have elevated levels of creatine phosphokinase (CK). In contrast to MLS, myopathy and axonal neuropathy are usually mild. Clinical neuromuscular manifestations include areflexia, sensory-motor neuropathy, and variable weakness and atrophy. Muscle biopsy and electromyography commonly demonstrate neuropathic changes and rarely myopathic alterations. ChAc usually slowly progresses over 15-30 years, but sudden death, presumably caused by seizures or autonomic involvement, may occur.

**Table 2:** Comparative Features

Disorder	ChAc	MLS	HDL2	PKAN
Gene	*VPS13A*	*XK*	*JPH3*	*PANK2*
Protein	Chorein	XK protein	Junctophilin-3	Panthothenate kinase 2
Inheritance	Autosomal recessive	X-linked	Autosomal dominant	Autosomal recessive
Acanthocytes	+++	+++	+/-	+/-
Serum CK (U/L)	300 - 3000	300 - 3000	Normal	Normal
Neuroimaging	Striatal atrophy	Striatal atrophy	Striatal and cortical atrophy	"Eye of the tiger" sign
Usual onset	20 - 30	25 - 60	20 - 40	Childhood
Chorea	+++	+++	+++	+++
Other movement disorders	Feeding and gait dystonia, tongue and lip biting, parkinsonism	Vocalizations	Dystonia, parkinsonism	Dystonia, parkinsonism, spasticity
Seizures	Generalized, partial-complex	Generalized	None	None
Neuromuscular manifestations	Areflexia, weakness, atrophy	Areflexia, weakness, atrophy	None	None
Cardiac manifestations	None	Atrial fibrillation, malignant arrhythmias, dilative cardiomyopathy	None	None

### McLeod Neuroacanthocytosis Syndrome

The McLeod blood group phenotype is defined by the absence of the Kx antigen and by weak expression of the Kell antigens, and may be incidentally detected on routine screening (Table 2) [4,24]. Most carriers of the McLeod blood group phenotype have acanthocytosis and elevated CK levels, and develop MLS over several decades [17,24]. Onset of neurological symptoms ranges from 25-60 years and disease duration may be more than 30 years, usually longer than in ChAc [17,24]. About one third of MLS patients present with chorea indistinguishable from that observed in HD [25], and most patients will develop chorea during the course of the disease. Additional involuntary movements include facial dyskinesias and vocalizations. In contrast to ChAc, only exceptional MLS patients have lip- or tongue-biting, dysphagia, dystonia, or parkinsonism [24]. Psychiatric manifestations including depression, schizophrenia-like psychosis and obsessive-compulsive disorder are frequent and may appear many years prior to the movement disorders [26]. A subset of MLS patients develops cognitive deficits, particularly in later disease stages. Generalized seizures occur in about half of the patients.

Elevated CK levels are almost always found, about half of the MLS patients develop muscle weakness and atrophy during the disease course, but severe weakness is only rarely observed [27]. However, MLS myopathy may predispose to rhabdomyolysis, in particular in the context of neuroleptic medication use [28]. Neuromuscular pathology shows sensory-motor axonal neuropathy, neurogenic muscle changes and variable signs of myopathy [27]. About 60% of MLS patients develop a cardiomyopathy manifesting with atrial fibrillation, malignant arrhythmias or dilated cardiomyopathy. Cardiac complications are a frequent cause of death, thus MLS patients and asymptomatic carriers of the McLeod blood group phenotype should have a cardiologic evaluation [24,29].

Some female heterozygotes show CNS manifestations related to MLS as well as corresponding neuropathological changes [8]. Reduction of striatal glucose uptake was demonstrated in asymptomatic female heterozygotes [26].

In addition, MLS may be part of a "contiguous gene syndrome" on the X chromosome including CGD, Duchenne muscular dystrophy or X-linked retinitis pigmentosa. This is of particular importance for boys with chronic granulomatous disease who survive into adulthood because of modern treatment modalities: they must be screened for the McLeod phenotype and should be regularly monitored for its complications.

### Huntington's Disease-like 2

HDL2 presents usually in young adulthood, but, as with HD, the age of onset is inversely related to the size of the trinucleotide repeat expansion (Table 2) [30]. Patients may develop psychiatric abnormalities as the initial manifestation, with later appearance of chorea, parkinsonism and dystonia [14]. The disease may evolve from chorea to a more bradykinetic, dystonic phenotype, or remain parkinsonian throughout the disease course, but unlike HD, this is not related to the size of the trinucleotide expansion. Unlike in ChAc and MLS, deep tendon reflexes are usually brisk; there are no peripheral nerve or muscle abnormalities, and seizures have not been reported. Acanthocytosis is found in about 10% of HDL2 patients and CK levels are normal. Neuroimaging reveals bilateral striatal atrophy, in particular of the caudate nucleus. In contrast to ChAc and MLS, generalized cortical atrophy may develop during the disease course. Neuropathologically, ubiquitin-immunoreactive intranuclear neuronal inclusions, similar to those seen in HD, are found [30].

### Pantothenate kinase-associated Neurodegeneration

PKAN is an autosomal recessive condition included in the group of disorders known as neurodegeneration with brain iron accumulation (NBIA). PKAN is the only NBIA in which acanthocytosis has been reported so far, and typically presents in childhood with rapid progression over 10 years (Table 2) [12]. Initial manifestations include orofacial and limb dystonia, choreoathetosis and spasticity. Lingual dystonia can be prominent, but is not specifically related to eating as in ChAc. Other speech difficulties specifically palilalia or dysarthria, are prominent features of PKAN [12]. Most patients develop pigmentary retinopathy and one third cognitive impairment. About 8 to 10% of PKAN patients have acanthocytosis, perhaps due to abnormalities of lipid synthesis [12]. Onset can be later, with more rigidity in atypical forms of PKAN [12], but the typical MRI findings of the "eye of the tiger" sign suggest the diagnosis.

## Aetiology

The genes responsible for the various NA syndromes have been identified.

ChAc is caused by various mutations of a 73 exon gene on chromosome 9, *VPS13A*, coding for chorein [9,10]. No obvious genotype-phenotype correlations have been observed. Chorein is implicated in intracellular protein sorting but its physiological functions are not yet known. Chorein is widely expressed throughout the brain and various internal organs. Almost all mutations to date appear to result in absence of chorein and there do not appear to be any partial manifestations of the disease, e.g. in heterozygous carriers.

MLS is caused by mutations of the *XK *gene encoding the XK protein, which carries the Kx erythrocyte antigen (11). Most pathogenic mutations are nonsense mutations or deletions predicting an absent or shortened XK protein lacking the Kell protein binding site. Although the exact function of the human XK protein is not elucidated, data from a *C. elegans *analogue of the *XK *gene suggest a possible role in apoptosis regulation [31]. The XK protein has ten transmembrane domains and probably has transport functions. In erythrocytes it is linked to the Kell protein via disulfide bonds. This complex carries the antigens of the Kell blood group, the third most important blood group system in humans. The Kx antigen (on XK) is absent in McLeod syndrome and expression of other Kell system antigens (on the Kell protein) is severely depressed [4,17]. In muscle, Kell and XK are not co-localized [32] and only XK but not Kell is present in neuronal tissue indicating different physiological functions of the two proteins in different tissues [33,34].

HDL2 is caused by expanded trinucleotide repeats of the junctophilin 3 gene (*JPH3*). As in HD, there is anticipation and the age of onset is inversely related to the size of the trinucleotide repeat expansion. Affected individuals have CTG/CAG repeat expansions of 41-59 triplets (normal population: 6-27).The expanded trinucleotide repeat in the *JPH3 *gene responsible for HDL2 causes both ubiquitinated intranuclear neuronal inclusions [30,35,36] and cytoplasmic mRNA inclusions [37]. There is evidence from cell culture studies that these latter inclusions are responsible for cell death [37]. *JPH3 *plays a role in junctional membrane structures, and may be involved in the regulation of calcium.

PKAN is caused by mutations of the pantothenate kinase 2 gene (*PANK2*) on chromosome 20p13. Truncating mutations are responsible for the majority of cases. PKAN catalyses the rate-limiting step in the synthesis of coenzyme A from vitamin B5 (pantothenate). The residual enzymatic activity correlates with the disease phenotype, as typical patients have no active enzyme but atypical patients with adult onset usually harbor *PANK2 *missense mutations [12]. Impaired lipid synthesis may account for the RBC acanthocytosis. Additional genes, responsible for further subtypes of NBIA, have recently been discovered. Nothing is yet known about the occurrence of acanthocytes in these.

## Diagnostic Considerations

The determination of acanthocytosis in peripheral blood smears may be negative in a standard setting and a negative screen does not exclude an NA syndrome [38]. Automated blood counts usually show an elevated number of hyperchromic erythrocytes. A more sensitive and specific method for the detection of acanthocytes uses a 1:1 dilution with physiological saline and phase contrast microscopy [39]. In contrast to the often elusive acanthocyte search, serum CK is elevated in most cases of ChAc and MLS.

ChAc patients have absent chorein expression in erythrocytes on Western blot (http://www.euro-hd.net/html/na/network/docs/chorein-wb-info.pdf) [40]. Confirmatory DNA analysis of the large VPS13A gene is difficult, due to the large gene size and heterogeneity of mutation sites [4-6,9,10], and is currently available only from a single commercial laboratory (http://www.mgz-muenchen.de). The diagnostic procedure of choice in MLS is the determination of absent Kx antigen and reduced Kell antigens on the erythrocytes in males and fluorescence absorbent cell sorting with Kell antigens in female heterozygotes. Analysis of the XK gene is confirmatory and offered by a number of academic laboratories.

**Figure 2:**
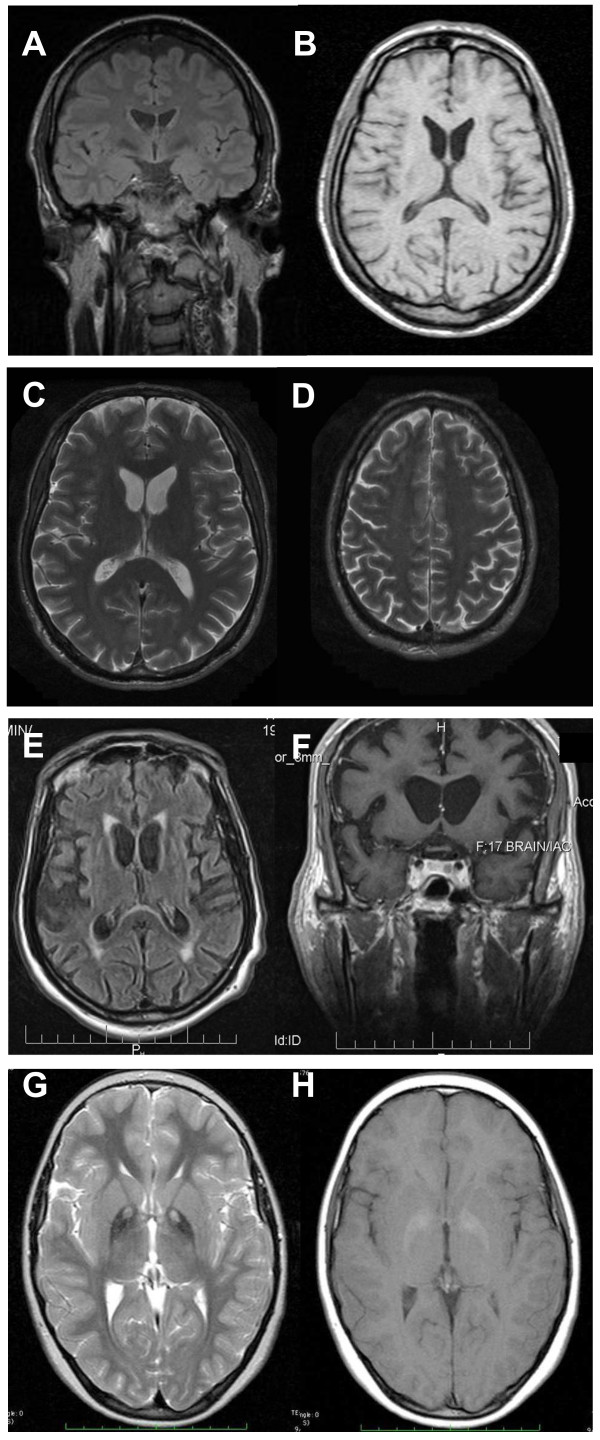
**Neuroimaging**. *ChAc. *Coronal FLAIR- (A) and axial T1-weighted (B) images demonstrate moderate atrophy of the caudate nucleus. *MLS. *Axial T2-weighted images demonstrate moderate atrophy of caudate nucleus and putamen (C) but no relevant cortical atrophy (D). *HDL2. *Axial FLAIR- (E) and coronal T1-weighted images (F) demonstrate atrophy of the caudate nucleus and the fronto-temporal cortex. In addition, FLAIR images show periventricular white matter hyperintensities (courtesy of Nora Chan, MD, UCLA, Los Angeles, USA). *PKAN. *T2-weighted fast spin echo (G) and T1-weighted (H) brain MRI scans from a child with PKAN demonstrating the "eye of the tiger" sign (courtesy of Susan J. Hayflick, MD, Oregon Health and Science University, Portland, Oregon, USA)

In ChAc and MLS, electroneurography may demonstrate sensorimotor axonal neuropathy whereas electromyography may show neurogenic as well as myopathic alterations. Electroencephalographic findings are not specific and may comprise normal findings, generalized slowing, focal slowing, and epileptiform discharges. Neuroradiologically, there is progressive striatal atrophy especially affecting the head of caudate nucleus and impaired striatal glucose metabolism similar to that seen in HD (Figure 2) [24,26]. Voxel-based morphometry of MRI scans in ChAc shows specific involvement of the head of the caudate nucleus [41,42]. Neurodegeneration in both core NA syndromes affects predominantly the caudate nucleus, putamen and globus pallidus. In ChAc, thalamus and substantia nigra are also involved. In contrast to HD, there is no significant cortical pathology [8,43-45]. Neuropathological findings consist of neuronal loss and gliosis of variable degree in these regions, but no inclusion bodies of any nature or other distinct neuropathological features have as yet been detected.

Cerebral MRI is often diagnostic in PKAN, and the diagnosis is confirmed by analysis of the *PANK2 *gene (Figure 2). Analysis of the *JPH3 *gene CTG expansion is useful in patients of African ancestry with suspected HDL2.

## Differential Diagnosis

The differential diagnosis of NA syndromes depends upon the presenting symptoms, which can be protean. Initial symptoms may suggest psychiatric disease, including schizophrenia, depression, obsessive-compulsive disorder, tics, Tourette's syndrome, cognitive impairment, personality change, or may consist of parkinsonism, chorea, dystonia, peripheral neuropathy, myopathy, cardiomyopathy, or seizures [1]. Persons harboring the McLeod blood group phenotype are sometimes identified upon blood donation, many years or even decades prior to development of neurological symptoms. An important constellation to consider McLeod testing is the diagnostic work-up of chronic granulomatous disease, particularly if X-linked. Both MLS and ChAc may be detected incidentally by the elevation of CK or liver enzymes. Recognition of the syndrome may avoid the need for invasive and non-diagnostic tests such as muscle, bone marrow, or liver biopsy.

ChAc, MLS, and HDL2 all present in young to middle adulthood, but MLS has usually the latest onset of neurological symptoms. PKAN typically presents during childhood or adolescence, although adult-onset has been reported, particularly in cases where mutations do not abolish all PANK2 enzyme activity.

Presence of self-mutilating lip and tongue biting, or other self-mutilation such as head-scratching or finger-biting is strongly suggestive of ChAc. Self-mutilation of a comparable nature may be seen in boys with Lesch-Nyhan syndrome, however in these cases the age of onset is very much younger. Patients with PKAN may also develop quite severe lingual dystonia, but it does not appear to be task-specific.

## Genetic Counseling

ChAc is unusual for an autosomal recessive disorder, since its presentation is in early to middle adulthood, when the patient's parents relatively unlikely choose to have further children. The chances of any sibling developing ChAc is 1:4. Children of affected subjects will inherit one mutant allele and will not be affected. MLS is X-linked, thus affected males will pass on the mutant X chromosome to their daughters, whose sons will have a 1:2 chance of developing MLS and daughters will have a 1:2 chance of being carriers. These female carrier heterozygotes rarely develop a neurological syndrome. PKAN is autosomal recessive, and siblings will have a 1:4 chance of developing disease. HDL2 is autosomal dominant, thus any child of an affected parent has a 1:2 chance of developing the disease. Siblings of an affected subject also have a 1:2 chance of being affected. Due to anticipation, related to expansion of the trinucleotide repeat, age of onset can be younger with successive generations. Since all genes are known, routine methods for prenatal testing can be applied.

## Management

So far no curative or disease-modifying treatments are available and management of the NA disorders is purely symptomatic. Recognition of treatable complications such as seizures, swallowing problems, and heart involvement is essential. Neuropsychiatric issues, particularly depression, can have a major impact upon quality of life, and these symptoms may be more amenable to pharmacotherapy than others. Dopamine antagonists or depleters such as tiapride, clozapine or tetrabenazine may ameliorate the hyperkinetic movement disorders. Seizures usually respond to standard anticonvulsants, including phenytoin and valproate, although lamotrigine and carbamazepine may worsen the involuntary movements [21]. Anticonvulsants may have the benefit of multiple parallel effects upon involuntary movements, psychiatric symptoms, and seizures. Cardiac complications in MLS need to be particularly considered and heart function should be monitored regularly. No patient with MLS to our knowledge has yet received a heart transplant, which could nevertheless be a management option.

Results of deep brain stimulation (DBS) in ChAc and MLS have been variable, and the optimal sites and preferred stimulation parameters remain to be determined [46-48]. Benefits have been observed with stimulation of both the ventro-oral posterior (Vop) thalamic nucleus and the GPi [46-48]. Thalamic stimulation in one ChAc patient resulted in a dramatic and sustained reduction of truncal spasms, but there was no clear effect upon dysarthria or on hypotonia. High frequency stimulation (130 Hz) of the GPi worsened speech and chorea, but improved dystonia, belching, dyskinetic breathing and tongue-biting. Low frequency stimulation (40Hz) improved chorea, but not dystonia. An ablative procedure may be a valuable surgical alternative if long-term implant management is likely to be problematic. In general, neurosurgical options should be considered experimental and must be tailored to individual cases.

Non-medical therapies with a multidisciplinary approach are often helpful. Evaluation by a speech therapist is essential to minimize problems due to dysphagia and weight loss. Dystonia of the lower face and tongue can result in severe tongue and lip self-mutilation in ChAc and may be ameliorated by a bite plate. Dystonic tongue protrusion whilst eating in ChAc may respond to local botulinum toxin injections into the genioglossus muscle, although this method has to be applied with caution due to possible mechanical obstruction of the airway and inefficient swallowing by paretic muscles. Placement of a feeding tube, temporarily or even continuously, including percutaneous gastrostomy, may be necessary to avoid nutritional compromise and to reduce the risk of aspiration. Physical and occupational therapists can assist with difficulties with gait, balance, and activities of daily living. Most importantly, extended and continuous multidisciplinary psychosocial support should be provided for the patients and their families.

## Prognosis

All NA disorders have a relentlessly progressive course and are eventually fatal. Sudden death may be due to seizure, or possibly autonomic dysfunction, but there may be gradually progressive, generalized debility, as seen in Huntington's or Parkinson's diseases, with patients succumbing to aspiration pneumonia or other systemic infections.

## Unresolved Questions

Much remains to be learned regarding the molecular mechanisms which cause neurodegeneration in the NA syndromes. The relationship between erythrocyte acanthocytosis and neurodegeneration is obscure, perhaps less so in PKAN. We hope that elucidation of molecular pathophysiology will lead to prevention and reversal of disease at the cellular level.

## Conclusions

NA syndromes must be included in the differential diagnosis of Huntington disease (HD). Their consideration is mandatory if HD genetic testing is negative. The NA syndromes have additional clinical characteristics such as epilepsy, peripheral neuropathy, cardiomyopathy (MLS) as well as orofacial dyskinesia, and feeding dystonia (ChAc). Paraclinical findings such acanthocytosis and elevated CK levels may be crucial to indicate the appropriate laboratory examination, in particular Kell blood group phenotyping and chorein Western blotting. Specific genetic testing may confirm the diagnosis. Management of NA syndromes is symptomatic, although life expectancy and quality of life may be augmented considerably by the appropriate measures.

## Competing interests

The authors declare that they have no competing interests.

## Authors' contributions

All authors contributed to the conception and design of the review. HHJ and RHW drafted the manuscript. All authors critically revised the manuscript and gave their final approval of the version to be published.
